# Tumor Environment-Responsive Hyaluronan Conjugated Zinc Protoporphyrin for Targeted Anticancer Photodynamic Therapy

**DOI:** 10.3390/jpm11020136

**Published:** 2021-02-17

**Authors:** Shanghui Gao, Rayhanul Islam, Jun Fang

**Affiliations:** Faculty of Pharmaceutical Sciences, Sojo University, Ikeda 4-22-1, Nishi-ku, Kumamoto 860-0082, Japan; g2031d01@m.sojo-u.ac.jp (S.G.); rayhanulislam88@gmail.com (R.I.)

**Keywords:** EPR effect, tumor targeting, photodynamic, hyaluronan, zinc protoporphyrin

## Abstract

Targeted tumor accumulation, tumor environment responsive drug release, and effective internalization are critical issues being considered in developing anticancer nanomedicine. In this context, we synthesized a tumor environment-responsive nanoprobe for anticancer photodynamic therapy (PDT) that is a hyaluronan conjugated zinc protoporphyrin via an ester bond (HA-es-ZnPP), and we examined its anticancer PDT effect both in vitro and in vivo. HA-es-ZnPP exhibits high water-solubility and forms micelles of ~40 nm in aqueous solutions. HA-es-ZnPP shows fluorescence quenching without apparent ^1^O_2_ generation under light irradiation because of micelle formation. However, ^1^O_2_ was extensively generated when the micelle is disrupted, and ZnPP is released. Compared to native ZnPP, HA-es-ZnPP showed lower but comparable intracellular uptake and cytotoxicity in cultured mouse C26 colon cancer cells; more importantly, light irradiation resulted in 10-time increased cytotoxicity, which is the PDT effect. In a mouse sarcoma S180 solid tumor model, HA-es-ZnPP as polymeric micelles exhibited a prolonged systemic circulation time and the consequent tumor-selective accumulation based on the enhanced permeability and retention (EPR) effect was evidenced. Consequently, a remarkable anticancer PDT effect was achieved using HA-es-ZnPP and a xenon light source, without apparent side effects. These findings suggest the potential of HA-es-ZnPP as a candidate anticancer nanomedicine for PDT.

## 1. Introduction

Targeted cancer therapy has been recognized as a key issue for achieving a successful anticancer outcome and is becoming a trend for developing anticancer drugs. In the past few decades, molecular target drugs designed to selectively inhibit the oncogene products and other molecules involved in tumor growth have attracted attention, and many such drugs get approved and used in clinics. However, the tumor heterogeneity, diversity, and frequent mutation of tumor-related molecules largely limit the application and availability of molecular target drugs [1,2]. To solve these problems and to increase the efficacy of molecular target drugs, personalized medicine or precision medicine has recently been proposed to intensely focus on the major genes/molecules in a single cancer patient [3]. This strategy is promising. However, there is still a long way to go, and extensive efforts and costs are needed to achieve success.

Recently, an alternative tumor-targeting strategy has drawn considerable interest, which targets the tumor tissues as a whole by using macromolecular drugs or nano-designed drugs, i.e., nanomedicine [4,5]. The principle of tumor tissue targeting is based on the unique phenomenon of tumor vasculature’s pathophysiological nature called the enhanced permeability and retention (EPR) effect [6,7,8,9]. Compared to normal blood vessels, tumor blood vessels have larger fenestration along with high vascular permeability due to the extensively produced vascular mediators such as nitric oxide (NO), bradykinin (BK), vascular endothelial growth factor (VEGF), etc. These vascular mediators contribute to macromolecules’ selective entry and accumulation in tumor tissues [7,8,9]. Furthermore, because of the dysfunctional lymphatic recovery system in tumors, macromolecules accumulated in tumor tissues could not be removed effectively, resulting in prolonged retention times [7,10]. Thus, by using nanomedicines, one could achieve much higher tumor concentrations of drugs than using conventional low molecular drugs. At the same time, a decreased distribution of nanomedicines in normal tissues could also be achieved because nanomedicines could not cross normal blood vessels, a benefit of their larger size, which is vastly different from conventional low molecular drugs that distribute indiscriminately in normal tissues. Consequently, a superior antitumor effect, as well as greatly lowered side effects, could be achieved for nanomedicines compared to conventional low molecular anticancer drugs [4,5,9,11,12]. To date, many anticancer nanomedicines have been developed, some are approved in the clinic, and more are in clinical trials or under preclinical evaluation [4,5,9].

Photodynamic therapy (PDT) is a promising therapeutic regimen for cancer. Singlet oxygen (^1^O_2_) and other reactive oxygen species (ROS) generated from photosensitizers (PS) upon light irradiation, are the effector molecules of PDT [13,14]. Namely, a PS is first administered, followed by irradiation using light with the appropriate wavelength, where PS in the tumor is excited, and the energy is transferred to molecular oxygen to generate ^1^O_2_. As a group of highly reactive molecules, ROS, including ^1^O_2,_ rapidly react with and damage different biomolecules, including proteins, DNA, and lipids, which leads to the apoptosis of tumor cells [14]. Now PDT is already in use for the treatment of some cancers, for example early-stage lung (bronchogenic) and superficial skin cancers [15,16,17]. However, to achieve the ideal PDT effect, PS must be delivered selectively into the tumor, otherwise an insufficient tumor concentration of PS will not generate enough ROS to kill cancer cells. Also, an unpreferable distribution may induce adverse side effects. For overcoming this drawback, EPR effect-based nano-designed PS is becoming popular for the targeted delivery of PS to tumor tissues. Many nano-platforms have been utilized to modify PS, including polymer conjugates, polymer micelles, liposomes, and antibody-drug conjugates [18,19,20,21]. In our group, by utilizing zinc protoporphyrin (ZnPP) and pyropheophorbide-a as PS, we have developed several polymeric micellar types of nanoprobes for PDT using different polymers, including polyethylene glycol (PEG), styrene maleic acid copolymer (SMA), *N*-(2-Hydroxypropyl) methacrylamide (HPMA) copolymer, and hyaluronan (HA), all of which showed a superior tumor-selective accumulation and thus therapeutic effect in different murine solid tumor models [22,23,24,25,26,27]. 

Among different polymers for preparing nanomedicines, HA as a natural polydisaccharide composed of alternative repeating units of *d*-glucuronic acid and *N*-acetyl-*d*-glucosamine, has attracted substantial interest as a biomaterial for medical applications as well as drug delivery systems because of its high biocompatibility, non-immunogenicity, non-toxicity, biodegradability, high water-solubility, and so on [28]. Many nanomedicines or nanoparticles using HA have been reported for the treatment of various diseases, including cancer and inflammatory diseases [29,30]. In our laboratory, we previously developed a HA conjugated ZnPP through an amide bond (HA-ZnPP), which formed micelles of 156 nm in aqueous solutions [27]. The micelle formation was relatively stable in physiological solution (e.g., circulation), and thus accumulated in tumors more selectively than free ZnPP [27]. Subsequently, after being taken up by tumor cells, the micelle formation was disrupted by lipid components in the cell membrane (i.e., lecithin), consequently fulfilling tumoricidal activity under xenon light irradiation [27]. However, compared to native ZnPP, the in vivo therapeutic effect of HA-ZnPP was not significantly improved, which is considered mostly due to its much lower rate of internalization than native ZnPP [27]. Namely, because of the stable uncleavable amide bond, HA-ZnPP could not release free ZnPP in tumors, thus remaining as a micelle, which quenched the activity of PS (ZnPP) and hindered ROS generation. It is thus of necessity and reasonable to further improve the therapeutic efficacy of HA-ZnPP by modulating the release of free ZnPP in tumor tissues.

In this context, tumor environment responsive release of free drugs from nanomedicines is known as a key issue for the design of anticancer nanomedicines. One focus is on the pH of tumor tissues that is weakly acidic (6~7) compared to the neutral pH (~7.4) of normal tissues [31,32]. Utilization of acidic pH-responsive chemical bonds such as the hydrazone bond is becoming a consensus for the development of anticancer nanomedicines, which have been proven to be an excellent strategy with high tumor targeting and therapeutic efficacy by many research groups and by using different polymers and drugs [12,33]. Another strategy focuses on the proteases in tumors. It is known that many tumors exhibit high levels of various proteinases, such as cathepsin B, cathepsin K, and esterase [13,34]. Thus, a protease/esterase-sensitive linker can be used to increase the tumor-specific release of free drugs, which has been proven as an effective approach in many studies [34,35]. As a proof of concept, in previous studies, we compared PEGylated ZnPP (PEG-ZnPP) with different chemical bonds (i.e., ester bond, ether bond, and amide bond) for the release properties under different conditions and in different tissues, including cancer [36]. The results clearly showed that PEG-ZnPP with ether bond and amide bond rarely released ZnPP derivatives in tumors as well as normal tissues, whereas, in the case of PEG-ZnPP with ester bond, cleaved ZnPP derivatives became the major components in the tumor but not in normal tissues except for liver due to its high esterase expression [36]; consequently, this tumor-responsive cleavage of the free drug resulted in a much stronger anticancer effect than those PEG-ZnPP with ether and amide bonds [36].

Along this line, in this study, we designed and synthesized a HA conjugated ZnPP via ester bond (HA-es-ZnPP) and investigated its potential as a nanoprobe for tumor-targeted PDT, especially focusing on its tumor environment-responsive release properties. Also, the tumor imaging capacity of HA-es-ZnPP toward anticancer theranostics was evaluated and is discussed here.

## 2. Materials and Methods

### 2.1. Materials

HA with a mean molecular weight of 30,000 (polydispersity index of 1.212) was purchased from MRC polysaccharide Corp., Ltd (Toyama, Japan). RPMI 1640 medium, 4-dimethylaminopyridine (DMAP) isoflurane, 3-(4,5-Dimethylthiazol-2-yl)-2,5-diphenyltetrazolium bromide (MTT), Tween 20, sodium dodecyl sulfate (SDS) and zinc acetate were purchased from Wako pure chemical (Osaka, Japan). Protoporphyrin IX (PP) and cetyltrimethylammonium bromide (CTA) were from Sigma-Aldrich Chemical (St. Louis, MO, USA). Water-soluble carbodiimide (WSC) was purchased from Dojindo Laboratory, Kumamoto, Japan. Additionally, 2,2,6,6-Tetramethyl-4-piperidone (4-oxo-TEMP) was purchased from Tokyo Chemical Industry (Tokyo, Japan). Esterase from porcine liver was purchased from Roche Diagnostics GmbH (Mannheim, Germany). The other reagents and solvents of a reagent grade were from commercial sources and were used without further purification.

### 2.2. Synthesis of HA Conjugated ZnPP via Ester Bond (HA-es-ZnPP)

Cetyltrimethylammonium salt of HA (CTA-HA) was first synthesized according to a previous report [37] with some modifications. Briefly, 110 mg of CTA bromide was dissolved in 1.5 mL of deionized water at 40 °C, to which an aqueous solution of HA (100 mg, 0.5% *w/v*) was added dropwise. The white precipitate thus formed was collected after centrifugation (10,000× *g*, 5 min), and washed three times with 40 mL of hot water, and finally dried under vacuum. 

ZnPP was prepared by inserting zinc into PP as described previously [38]. In brief, a 10-time molar excess of zinc acetate was added to PP in dimethyl sulfoxide (DMSO) and stirred at 60 °C for 24 h. After cooling, cold deionized water was added 5-fold to the volume of DMSO to precipitate the product ZnPP, which was purified by centrifugation to remove excess zinc acetate and washed thrice with cold deionized water. 

**Figure 1:**
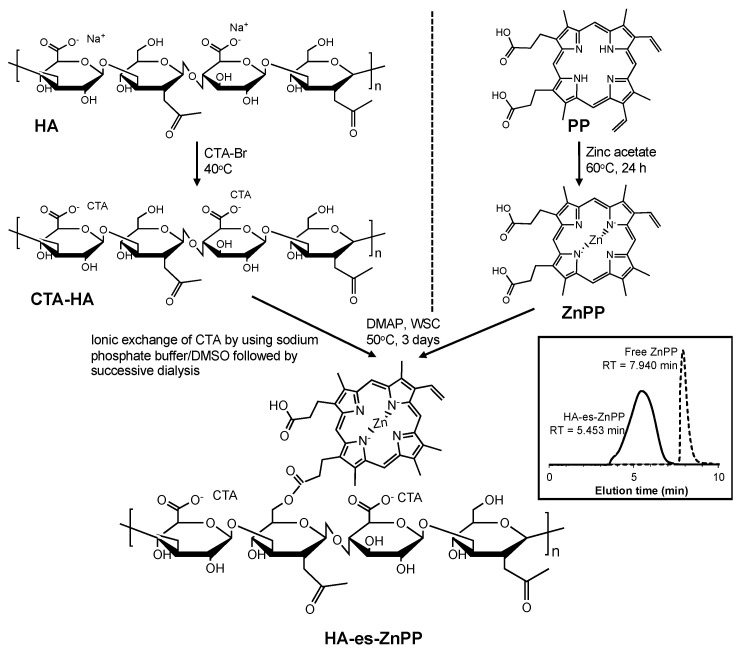
Synthesizing protocol of hyaluronan (HA) conjugated ZnPP through ester bond (HA-es-ZnPP). Inset shows the gel permeation chromatograph of HA-es-ZnPP and free ZnPP, by using a high-performance liquid chromatography (HPLC) system with an Asahipak GF-310 HQ column.

CTA-HA obtained as described above was dissolved in 20 mL of DMSO, and 10 mg of ZnPP that was dissolved in 1 mL of DMSO was added dropwise, then DMAP (500 mg) and WSC (500 mg) were added to the reaction mixture. The reaction was continued for 3 days at 50 °C. The solution was then subjected to dialysis using a dialysis bag with an average cutoff of 8000 (Wako) against a 1:1 (*v/v*) sodium phosphate buffer (0.3 M, pH = 7.4): DMSO mixture for 1 day, then against 5% sodium bicarbonate for 1 day, and finally against deionized water for 1 day. Each solvent was changed every 8 h. The solution was then lyophilized to give a brown powder. Figure 1 shows the synthetic scheme of HA-es-ZnPP. During the reaction, high-performance liquid chromatography (HPLC) was performed to monitor the reaction by using LC-2000Plus series HPLC system (JASCO, Tokyo, Japan) with an Asahipak GF-310 HQ column (7.5 × 300 mm) (Showa Denko, Tokyo, Japan). Using a mobile phase of 70% methanol/30% DMSO with 0.001% trifluoroacetic acid (Wako) at a flow rate of 0.8 mL/min, the eluate was monitored at 415 nm for ZnPP.

### 2.3. Characterization of HA-es-ZnPP

#### 2.3.1. UV–Visible Spectrophotometry and Fluorescence Spectroscopy

UV/vis absorption spectra and fluorescence spectrum of ZnPP and HA-es-ZnPP were measured by a spectrophotometer (Hitachi U-3900, Tokyo, Japan), and a spectrofluorometer model FP-6600 (Jasco Corp., Tokyo, Japan), respectively. For spectroscopy, the sample solution was excited at 420 nm, and emissions from 550 to 700 nm were recorded.

#### 2.3.2. Determination of the ZnPP Content in HA-es-ZnPP

Standard ZnPP solutions at 0.05–1 mg/mL and HA-es-ZnPP (1 mg/mL) were prepared in DMSO. The standard curve of ZnPP was then created by measuring the absorbance of samples at 421 nm (absorption maximum of ZnPP), and the content of ZnPP in HA-es-ZnPP was calculated according to the standard curve.

#### 2.3.3. Dynamic Light Scattering (DLS) and Zeta Potential

HA-es-ZnPP was dissolved in phosphate-buffered saline (PBS) at 5 mg/mL and filtered through a 0.45 μm filter. The particle size and surface charge (zeta potential) were measured by using an electrophoretic light-scattering analyzer (ELS-Z2, Otsuka Electronics Co., Ltd., Osaka, Japan).

### 2.4. Release of ZnPP from HA-es-ZnPP

To investigate the release of ZnPP from HA-es-ZnPP conjugate, HA-es-ZnPP of 5 mg/mL was dissolved in a 0.2 M sodium phosphate buffer (PB) of different pHs (i.e., 6.5, 7.4, 8.5) with 0.5% Tween 20. For the study of an enzymatic effect on drug release, porcine liver esterase solution (10 units/ml) was added to a HA-es-ZnPP solution in a pH 7.4 PB buffer with 0.5% Tween 20. In some experiments, HA-es-ZnPP was added to 100% fetal bovine serum (FBS) or supernatant of tumor homogenate from mouse sarcoma S180 as described below, to the final concentration of 5 mg/mL. All samples (each of 5 mL) were then placed in dialysis tubes with a cutoff of ~8000 Da and were dialyzed against PBS (25 mL) with shaking (1 Hz) at 37 °C. At each indicated time point, 0.1 mL of the solution outside the dialysis bag was collected, which was diluted by 2 mL DMSO followed by fluorescence measurement as described above. The amount of released ZnPP was quantified according to a standard curve using standard ZnPP samples.

#### O_2_ Generation from HA-es-ZnPP as Measured by Electron Spin Resonance (ESR) Spectroscopy

HA-es-ZnPP (40 μg/mL ZnPP equivalent) was dissolved in PBS with/without 0.5% Tween 20, to which 20 mM of 4-oxo-TEMP (spin trapping agent) was added. Samples were placed in a flat quartz cell (Labotec, Tokyo, Japan), and then subjected to light irradiation, using a xenon light source (MAX-303; Asahi Spectra Co. Ltd., Tokyo, Japan) at a wavelength of 400–700 nm. The ESR spectra were recorded by using a JEOL JES FA-100 spectrometer (Tokyo, Japan) at 25 °C, with a microwave power of 1.0 mW, amplitude of 100 kHz, and field modulation width of 0.1 mT.

### 2.5. Intracellular Uptake of HA-es-ZnPP

Mouse colon cancer C26 cells were seeded in a 12-well plate (5 × 10^5^ cells/well). After overnight pre-incubation, 20 μg/mL (ZnPP equivalent) of ZnPP dissolved in DMSO, or HA-es-ZnPP dissolved in PBS was added to the cells. After washing thrice with cold PBS at the scheduled time point, the cells were harvested, collected, and sonicated (30 W, 30 s, Dr.Hielscher, UP50H homogenizer, tip drip type) in ethanol on ice, followed by centrifugation (10,000× *g*, 5 min). The amount of ZnPP/HA-es-ZnPP in the supernatant was quantified by measuring fluorescence intensity as described above.

### 2.6. In Vitro Cytotoxicity Assay

Mouse colon cancer C26 cells were maintained in RPMI-1640 medium supplemented with 10% fetal bovine serum (FBS, Nichirei Biosciences Inc.,Tokyo, Japan) under 5% CO_2_/air at 37 °C. For cytotoxicity assay. Cells were seeded in 96-well plates (3000 cells/well). After overnight incubation, HA-es-ZnPP or free ZnPP of different concentrations was added, and the cells were cultured for 24 h. Irradiation was then carried out by using fluorescent blue light with a peak emission at 420 nm (TL-D; Philips, Eindhoven, The Netherland), at a total energy of 1.0 J/cm^2^ (3.3 mW/ cm^2^, 5 min). After a further 24 h of culture, cell viability was measured by MTT assay.

### 2.7. In Vivo Tissue Distribution of HA-es-ZnPP

Male ddY mice that were 6 weeks old were purchased from SLC Inc., Shizuoka, Japan. All animals were maintained at 22 ± 1 °C and a 55% ± 5% relative humidity, with a 12-h light/dark cycle. All of the experiments were approved by the Animal Ethics Committees of Sojo University (no. 2020-P-009, approved on April 01, 2020) and were carried out according to the Laboratory Protocol for Animal Handling of Sojo University.

A mouse sarcoma S180 solid tumor model was established by injecting S180 cells (2 × 10^6^) that were maintained in the ascitic form in the ddY mice into the dorsal skin of the ddY mice. After 10-12 days of tumor cell inoculation, when the tumor grew to ~10 mm in diameter, HA-es-ZnPP (5 mg/kg, ZnPP equivalent) dissolved in physiological saline was injected intravenously (i.v.) via the tail vein. For comparison, free ZnPP (5 mg/kg) that was dissolved in 0.01 N NaOH with 10% DMSO was injected i.v. At 24 h after i.v. injection, mice were killed, and tumors as well as normal tissues (e.g., liver, spleen, kidney, etc.) were collected. Tissues were first subjected to fluorescence imaging using IVIS XR (Caliper Life Science, Hopkinton, MA, USA) with Ex-420 nm/Em-DsRed. Then, to 100 mg of each tissue, 1 mL of DMSO was added, followed by homogenization. After centrifugation (10,000× *g*, 10 min), the extracted HA-es-ZnPP in the supernatant was quantified by fluorescence intensity (Ex 420 nm/Em 630 nm).

### 2.8. In Vivo Antitumor Activity of HA-es-ZnPP

Mouse sarcoma S180 solid tumor model, as described above, was used in this assay. At 7–10 days after tumor inoculation, when the diameters of the tumors became 8–10 mm, HA-es-ZnPP dissolved in physiological saline at indicated concentrations was administered i.v. Physiological saline was used for control mice. At 24 and 48 h after injection of HA-es-ZnPP, irradiation to the tumors was carried out using xenon light (MAX-303; Asahi Spectra) at 400–700 nm for 5 min (36 J/cm^2^), under anesthesia with isoflurane gas. The tumor volume (mm^3^), which was calculated as (W^2^ × L)/2 by measuring the width (W) and length (L) of the tumor, and body weight of mice were recorded every 2–3 days during the study period. 

### 2.9. Statistical Analyses

All data were expressed as means ± SD. Statistical analyses were performed by using GraphPad Prism 8.0 (GraphPad Software, Inc. La Jolla, CA, USA). Data were analyzed by using ANOVA followed by the Bonferroni correction for multiple comparisons (*p*-value was corrected by multiplying it by the number of comparisons), and unpaired Student’s *t*-test for dual comparisons. A difference was considered to be statistically significant when *p* < 0.05.

## 3. Results

### 3.1. Synthesis and Characterization of HA-es-ZnPP

HA-es-ZnPP was synthesized by using the carboxyl group of ZnPP and hydroxyl group of HA, which forms an ester bond (Figure 1). Gel permeation chromatograph using a HPLC system with an Asahipak GF-310 HQ column as described above, showed a single peak of the product HA-es-ZnPP with a retention time of 5.453 min, whereas ZnPP exhibited a retention time of 7.940 min (inset of Figure 1), suggesting the completion of the conjugation. 

**Figure 2:**
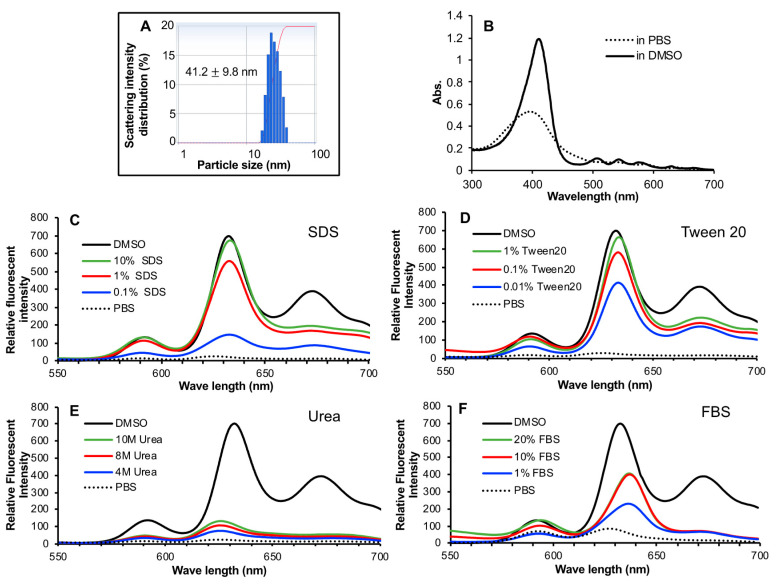
Micelle formation of HA-es-ZnPP. HA-es-ZnPP showed a hydrodynamic diameter in physiological saline of 41.2 nm, as measured by DLS (**A**). The micelle formation was supported by decreased absorbance as seen in UV/vis spectra (**B**), and more importantly, fluorescence quenching, the fluorescence intensity of HA-es-ZnPP in PBS was almost undetectable compared to that in DMSO (**C**,**D**). The micelle formation could be effectively disrupted by detergent SDS (**C**) and Tween 20 (**D**), as evidenced by increased fluorescence, but it could not be affected by urea (**E**). Increased fluorescence intensity/disruption of micelles was also found in the presence of FBS (**F**). See text for details.

The resulted HA-es-ZnPP had a ZnPP loading of 10% (wt/wt, 5–6 ZnPP units in one HA molecule) based on absorbance of ZnPP at 421 nm in DMSO, and showed very good water-solubility (>20 mg/mL). In an aqueous solution, HA-es-ZnPP showed an average particle size of 41.2 nm by DLS with a polydispersity index of 0.012 (Figure 2A), and the zeta potential of HA-es-ZnPP was −37.12 mV. These results indicated the micelle formation of HA-es-ZnPP in which ZnPP creates the hydrophobic core and hydrophilic HA forms the outer surface, as similarly seen in other examples of polymeric ZnPP reported in our previous studies [24,26,27,38,39,40]. The decreased absorbance of ZnPP supported the micelle formation in PBS compared to that in DMSO (Figure 2B). More pieces of evidence were obtained by quenching of fluorescence from HA-es-ZnPP in an aqueous solution (i.e., PBS) compared to the strong fluorescence of DMSO solution of HA-es-ZnPP (Figure 2C,D). The micelle formation would be disrupted by detergent SDS and Tween 20 in a concentration-dependent manner, as evidenced by the increased fluorescence (Figure 2C,D). However, no apparent change was found in the presence of urea (Figure 2E). These findings suggested the micelle was formed mostly by the hydrophobic interactions, but hydrogen bond is not involved. Moreover, increased fluorescence intensity was found in the presence of 10% FBS, but an increase of FBS concentration did not result in a further increase of fluorescence intensity (Figure 2E), suggesting that micelle formation could be partially disrupted in the circulation.

### 3.2. Release Profiles of HA-es-ZnPP

**Figure 3:**
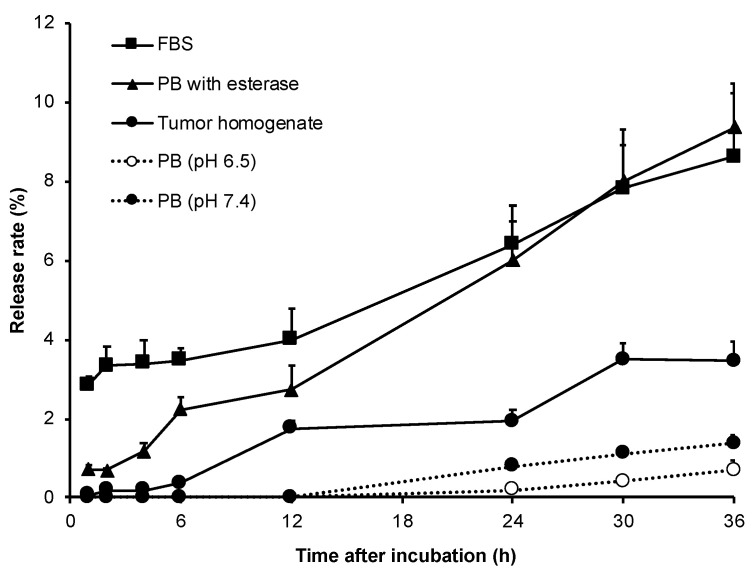
ZnPP release from HA-es-ZnPP at different conditions. In sodium phosphate buffer, almost no ZnPP was released from the conjugate at both physiological pH and weak acidic pH up to 12 h. Much rapid release of ZnPP occurred in presence of either esterase or FBS having esterase activities. A relatively high level of ZnPP release was also found in tumor homogenate.

As shown in Figure 3, in PB of physiological pH (7.4) and weak acidic pH (6.5), almost no release of ZnPP from HA-es-ZnPP was found till 12 h, while the release reached to 0.7–1.4% in 36 h. However, in the presence of esterase, a significant increase of ZnPP release was triggered; almost 10% of ZnPP was released in 36 h (Figure 3), which is another side supported that ZnPP was conjugated to HA via an ester bond. In parallel with this finding, in 100% FBS, which is known to exhibit esterase activity [41], increased release was observed, which were 6.5% and 8.6% in 24 h and 48 h, respectively (Figure 3). More importantly, when HA-es-ZnPP was incubated in the homogenate of mouse solid tumor (S180), relatively high levels of ZnPP release were achieved, e.g., 3.5% after 36 h incubation which is about 3-time higher that in PB (Figure 3), suggesting a preferable release behavior of HA-es-ZnPP in the tumor site.

### 3.3. Generation of ^1^O_2_ from HA-es-ZnPP after Light Irradiation

**Figure 4:**
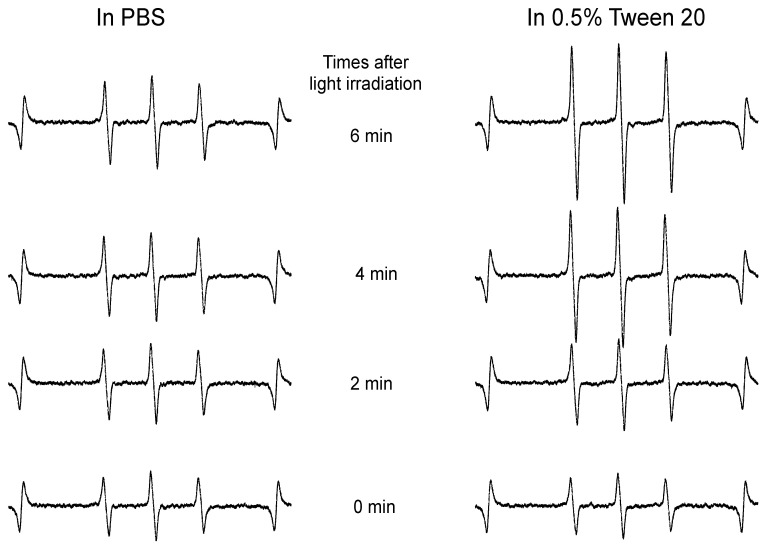
Electron Spin Resonance (ESR) spectra of HA-es-ZnPP in phosphate-buffered saline (PBS) in the presence/absence of Tween 20 under light irradiation. ^1^O_2_ generation from HA-es-ZnPP was captured by 4-oxo-TEMP, and triplet 4-oxo-TEMP signal as the indicator of ^1^O_2_ was detected by ESR spectra. In the presence of Tween 20, a relatively high level of ^1^O_2_ generation was observed in an irradiation time-dependent manner, whereas no ^1^O_2_ generation occurred in PBS without Tween 20, in which HA-es-ZnPP behaves as micelles.

The capacity of HA-es-ZnPP to generate ^1^O_2_ was evaluated by means of ESR spectroscopy under light irradiation using a xenon light source. Similar to our previous studies of polymeric ZnPP using HPMA copolymer [24,27], as the micelle form in aqueous solution (i.e., PBS), no apparent generation of ^1^O_2_ was observed even after 6 min of light irradiation (Figure 4). However, when micelle formation was disrupted in the presence of 0.5% of Tween 20, a strong ESR signal of ^1^O_2_ was found in an irradiation time-dependent manner (Figure 4). These findings agree with those of fluorescence spectroscopy showing fluorescence quenching in PBS and appearance of fluorescence in Tween 20 (Figure 2D). 

### 3.4. Intracellular Uptake of HA-es-ZnPP

**Figure 5:**
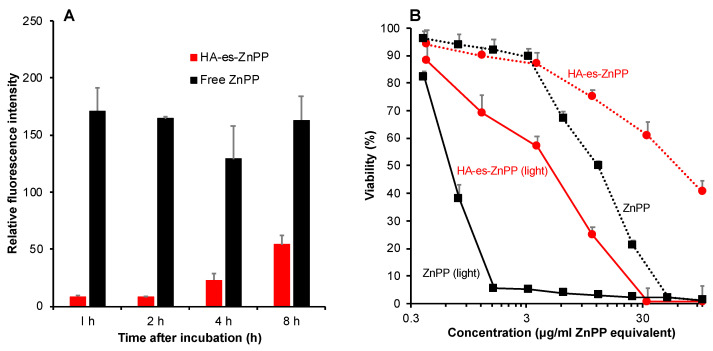
Intracellular uptake (**A**) and in vitro cytotoxicity (**B**) of HA-es-ZnPP in C26 colon cancer cells. (**A**), free ZnPP or HA-es-ZnPP (20 µg/mL ZnPP equivalent) was added to C26 cells for the indicated time. The intracellular ZnPP or HA-es-ZnPP was detected and quantified by measuring fluorescence intensity. (**B**), free ZnPP or HA-es-ZnPP at different concentrations was added into the cells, and the cells were treated for 48 h. In a separate plate, cells were irradiated by using fluorescent blue light (1.0 J/cm^2^, 5 min irradiation) at 24 h after HA-es-ZnPP treatment followed by further 24 h incubation. The cell viability was then measured by MTT assay. Data are mean ± SD.

Free ZnPP was rapidly and extensively internalized into C26 colon cancer cells as early as 1 h after incubation and maintained similar levels for up to 8 h (Figure 5A). Compared to free ZnPP, HA-es-ZnPP exhibited a slower but constant uptake by C26 cells, at 8 h after incubation, the intracellular fluorescence from internalized ZnPP in cells treated with HA-es-ZnPP was one-third of that in free ZnPP treated cells (Figure 5A).

### 3.5. In Vitro Cytotoxicity of HA-es-ZnPP

*In vitro* cytotoxicity of HA-es-ZnPP was examined in a C26 colon cancer cell line. As shown in Figure 5B, without light irradiation, ZnPP per se showed a cell-killing effect with an inhibitory concentration (IC_50_) of 12.5 μg/mL, whereas a more than 15-time increased cytotoxicity was achieved after light irradiation, indicating a remarkable PDT effect. Compared to free ZnPP, HA-es-ZnPP itself exhibited lower cytotoxicity with an IC_50_ of 50 μg/ml (ZnPP equivalent). However, after light irradiation, significantly increased cytotoxicity (IC_50_ of 4.5 μg/mL) was observed, which is 10-fold higher than that of HA-es-ZnPP per se (Figure 5B). These results are parallel with the findings of intracellular uptake shown in Figure 5B.

### 3.6. In Vivo Tissue Distribution of HA-es-ZnPP after I.V. Injection

**Figure 6:**
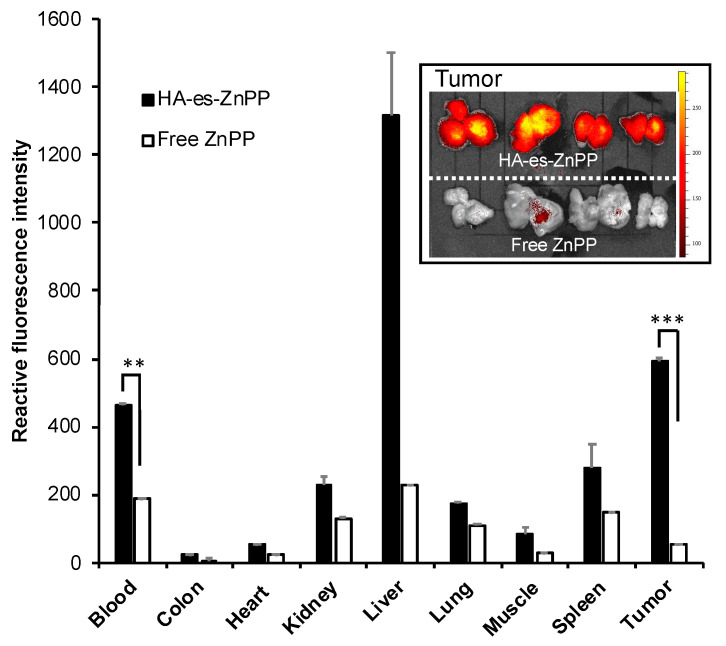
*In vivo* tissue distribution of HA-es-ZnPP in S180 solid tumor-bearing ddY mice. HA-es-ZnPP or free ZnPP was injected i.v. in the mice. After 24 h, mice were killed, and tissues, including tumors were collected. Tissue samples were homogenated using DMSO, and extracted HA-es-ZnPP or ZnPP in the supernatant was quantified by fluorescence spectroscopy. Inset shows the fluorescence images of mice-tumors treated with free ZnPP or HA-es-ZnPP. The tumors were cut in the middle of tumor nodules, and the cross-sectional views were shown. Data are mean ± SD. **, *p* < 0.01; ***, *p* < 0.001.

**Figure 7:**
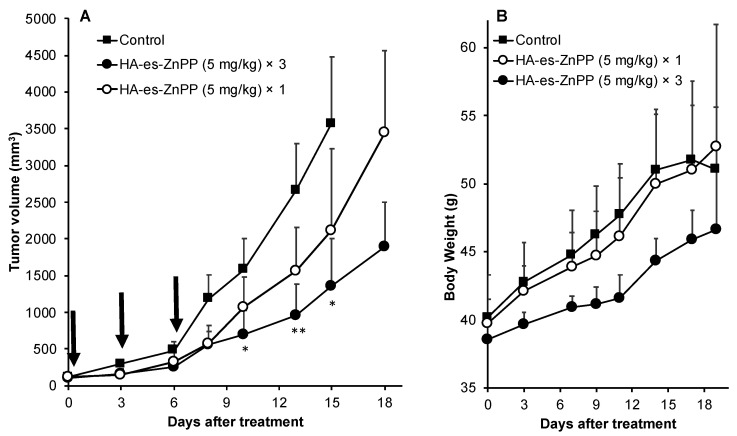
*In vivo* therapeutic effect of HA-es-ZnPP based PDT. In a mouse sarcoma S180 solid tumor model, HA-es-ZnPP (5 mg/kg, ZnPP equivalent) was injected i.v. when the tumor grew to 8–10 mm in diameter. At 24 and 48 h after injection of HA-es-ZnPP, irradiation to the tumors was carried out using xenon light (MAX-303; Asahi Spectra) at 400–700 nm for 5 min (36 J/cm^2^). This treatment protocol was carried out 1 time or 3 times in different groups. The tumor volume (**A**) and body weight (**B**) of mice were recorded every 2–3 days during the study period. Arrows indicate the PDT treatments. Data are mean ± SD. *, *p* < 0.05; **, *p* < 0.01.

Tissue distribution of HA-es-ZnPP in mice bearing sarcoma S180 solid tumor at 24 h after i.v. administration of HA-es-ZnPP was shown in Figure 6. Compared with native ZnPP which showed very low or negligible distribution in all tested tissues, including tumor (Figure 7A), HA-es-ZnPP exhibited a significantly prolonged circulation time, while the blood concentration of HA-es-ZnPP was 2.5-times more than that of ZnPP (Figure 7A). 

More importantly, tumor accumulation of HA-es-ZnPP was 10-times higher than that of free ZnPP (Figure 7A). Moreover, the amount of HA-es-ZnPP in the tumor was higher than those in most of the normal tissues except the liver, which is rich in reticuloendothelial systems and is the primary organ for the protoporphrin metabolism [24,39,42]. These findings suggest a preferentially targeted delivery of HA-es-ZnPP in the tumor by taking advantage of the EPR effect. The tumor-selective delivery of HA-es-ZnPP was also visualized and confirmed by using fluorescence imaging involving detecting the fluorescence from ZnPP, during which much-intensified fluorescence was detected in tumors from mice treated by HA-es-ZnPP compared to those in free ZnPP treated mice (inset of Figure 6). In addition, the pictures shown in the inset of Figure 6 were the cross-sectional view of tumors, which clearly showed a strong fluorescence of HA-es-ZnPP in the core of the tumor (inset of Figure 6). 

### 3.7. In Vivo Antitumor Effect of PDT Using HA-es-ZnPP 

The in vivo therapeutic effect of PDT using HA-es-ZnPP was evaluated in a mouse sarcoma S180 solid tumor model. Irradiation was carried out twice at 24 h and 48 h after HA-es-ZnPP administration using a xenon light source, at 36 J/cm^2^, which is a relatively low irradiation dose [25]. By using this protocol, a dose-dependent PDT effect was observed, i.e., one round of treatment resulted in a slight suppression of tumor growth, whereas significantly delayed tumor growth was found when the treatment protocol was repeated 3 times (Figure 7A). Moreover, three injections of HA-es-ZnPP alone without light irradiation, or light irradiation alone without HA-es-ZnPP did not show apparent tumor suppression (Supplemental data Figure S1). In addition, a weight gain but no loss of body weight was observed in all groups during the treatment (Figure 7B).

## 4. Discussion

For the development of anticancer nanomedicine, several critical issues must be considered. The first necessary step is the EPR effect-based tumor accumulation. For this aim, nanomedicine must be stable enough in circulation to ensure there is a sufficient circulation time that is essential for achieving the EPR effect. Otherwise, disruption of unstable nano-formulation in circulation will result in similar behaviors to those of small molecular drugs [10,12,43]. In this context, HA-es-ZnPP behaves as micelles of ~40 nm (Figure 2A) and maintains the stable micelle formation in an aqueous solution as evidenced by the fluorescence quenching (Figure 2C,D). In the presence of FBS, though fluorescence quenching was partly liberated, complete disruption of micelles was not seen (Figure 2F), indicating HA-es-ZnPP could maintain at least a partially stable micelle formation in circulation. As a consequence, relatively large amount of HA-es-ZnPP remained in circulation compared to ZnPP at 24 h after i.v. injection, indicating a much longer blood half-life of HA-es-ZnPP than native ZnPP (Figure 6). In accordance with this finding, HA-es-ZnPP remarkably accumulated in the tumor, which showed a 10-times higher concentration than that of native ZnPP (Figure 6). Moreover, when we cut the tumors, we found stronger fluorescence (HA-es-ZnPP) in the tumor center than in the peripheral area (inset of Figure 6). It is known that the core of tumors is usually poor in blood vessels with many necroses, while the peripheral areas are actively growing parts with rich vasculature. This finding suggests that HA-es-ZnPP could penetrate tumor tissue effectively after extravasation from tumor blood vessels, which is probably due to its relatively small size (i.e., 40 nm) as demonstrated by Kataoka’s group; smaller nano micelles (i.e., 30 nm) penetrate much deeper into tumor tissues than larger micelles (i.e., 80 nm) [44]. Together, these findings strongly indicate the preferable properties of HA-es-ZnPP as a nanomedicine to target tumors by the EPR effect.

However, nanomedicine should quickly and effectively unload active pharmaceutical ingredients (API) to fulfill a therapeutical effect after reaching the tumor in a way that benefitted from the EPR effect. Otherwise, too stable nanomedicines with little API release in tumor sites will suffer from insufficient therapeutic activity [10,12,45]. In this context, nanomedicines with tumor environment responsive linkers have attracted more and more attention. Many strategies have been developed by utilizing, as examples, acidic pH-sensitive bond (e.g., hydrazone bond) or a protease cleavable peptide linker [31,32,34,35], as described above in “Introduction”. Besides, other strategies such as using magnetically responsive peptide [46], thermosensitive nano-platform [47], and Arg-Gly-Asp (RGD) linker [48] have been challenged which showed promising results. In the present study, we focused on the relatively high esterase activity in tumors [36], and designed a HA conjugated ZnPP with ester bond, i.e., HA-es-ZnPP. As expected, the covalent bond of HA-es-ZnPP ensures little release of ZnPP in water solutions, but ZnPP release could be triggered in the presence of esterase as well as FBS (Figure 3), indicating that HA-es-ZnPP will release ZnPP in circulation and more importantly, in tumor tissues. However, it should also be noted that the release of ZnPP in FBS was less than 10% in 36 h (Figure 3), which again supported that HA-es-ZnPP behaves as a relatively stable nano-micelles in circulation.

Intracellular uptake of API released from nanomedicines is also a critical matter for achieving a satisfactory anticancer effect, because in most cases, entering the cell and accessing the cellular components are necessary for anticancer drugs’ actions. Regarding this issue, low molecular weight hydrophobic drugs usually show rapid and extensive internalization. In contrast, most nano-drugs modified with hydrophilic polymers such as PEG suffer from the low cellular absorption and thus therapeutic effect, as known as “PEG dilemma” [49]. Our previous studies also showed that polymer conjugated ZnPP with covalent bonds exhibited much lower intracellular uptake than the corresponding native drugs [24,50]. Given this situation, the release of API from nanomedicine in the tumor site is an important matter of concern for developing anticancer nanomedicines, as described above. By use of the ester bond, in this study, we found that although the intracellular uptake of HA-es-ZnPP was slower and lower than free ZnPP, the uptake increased gradually in a time-dependent manner, which reached a level of one-third of that of ZnPP after 8 h (Figure 5). This result is partly parallel with the release profiles in the presence of FBS (Figure 3), suggesting that effective internalization of active drug (ZnPP) could be achieved in HA-es-ZnPP by benefiting from the esterase-dependent cleavage of ester bond and release of ZnPP. In addition, it is well known that CD44, the primary membrane receptor of HA, is highly expressed in many tumor cells [51]; thus, the CD44 mediated endocytosis may also contribute to the internalization of HA-es-ZnPP. As a consequence, HA-es-ZnPP alone showed relatively potent cytotoxicity to tested cancer cells with a IC_50_ of 50 µg/mL, which is one fourth the cytotoxicity of free ZnPP (12.5 µg/mL) (Figure 5B), and is much more potent than polymer conjugate of ZnPP using uncleavable bonds previously developed in our laboratory (i.e., IC_50_ > 100 µg/mL for HPMA conjugated ZnPP) [24]. 

More importantly, after light irradiation, the cytotoxicities of both free ZnPP and HA-es-ZnPP were remarkably (10–15 times) augmented (Figure 5B). The increased cytotoxicity was attributed to the generation of ^1^O_2_, i.e., PDT effect, which was confirmed in ESR measurement as indicated by the time-dependent intensified spin-polarized triplet signal (Figure 4). One concern about anticancer PDT is the off-target generation of toxic ^1^O_2_, namely, generation of ^1^O_2_ in normal tissues will induce adverse side effects. This could be substantially improved by nano-designed PS to target tumor tissue by taking advantage of the EPR effect as discussed in “Introductions”. In this regard, HA-es-ZnPP, as a micellar type nanomedicine, exhibited much higher accumulation in tumors than most normal tissues, which could not be seen in case of small molecule free ZnPP (Figure 6). In addition, interestingly, we found that HA-es-ZnPP in micellar form did not emit ^1^O_2_ as well as fluorescence under light irradiation (Figure 2 and Figure 4). This was probably due to a π–π stacked structure of ZnPP in the core of micelles, in which excited fluorochrome dissipates the energy [24]. This property will further ensure the safety of anticancer PDT using HA-es-ZnPP. Namely, in circulation HA-es-ZnPP behaves mostly as micelles with relative stability, so no or largely reduced ^1^O_2_ will be generated under ambient light. During circulation, HA-es-ZnPP will accumulate and retain in the tumor due to the EPR effect, during which micelles will be gradually disrupted and free ZnPP is released and is then taken up by tumor cells. The following light irradiation will thus trigger the generation of ^1^O_2_ mostly in the tumor to fulfill the anticancer effect, whereas less side effects to normal tissues and organs will be induced. Thus, the PDT using HA-es-ZnPP will confer a superior therapeutic effect over conventional PDT using low molecular weight PS as well the improved safety of the treatment and quality of life in patients. As expected, we found significant suppression of tumor growth by PDT using HA-es-ZnPP (Figure 7A), whereas we did not observe body weight loss in this treatment regimen that indicates no apparent side effects (Figure 7B). It should be noted that the dose of HA-es-ZnPP (i.e., 5 mg/kg) and intensity of light irradiation (36 J/cm^2^) are relatively low compared to many other studies [25]; we thus believe the therapeutic effect could be optimized by modulating the dosing/irradiation regimen which will be investigated in future studies.

Compared to other polymeric PS, HA-es-ZnPP also potentially has some other advantages beyond the PDT effect. Besides the EPR effect-based tumor targeting and CD44-driven tumor targeting as described above [52], HA could also work in a tumor microenvironment to trigger the reprogramming of tumor-associated macrophages towards anti-tumor M1 type macrophages. Thus, a synergistic anticancer effect could be achieved using HA-based nanomedicine [53]. Moreover, because of the extensive tumor accumulation of HA-es-ZnPP (Figure 6), it may become possible to visualize tumors, especially tiny metastatic tumor nodules and disseminated cancer by using fluorescence imaging, as indicated in Figure 6 (inset). Namely, HA-es-PDT could be a theranostic nanoprobe for PDT as well as for photodynamic diagnosis, which warrants further investigations.

Taken together, in this study, we successfully synthesized a HA conjugated ZnPP via an ester bond, (Figure 1), which exhibits high water-solubility and forms micelles in aqueous solutions (Figure 2). As micellar formation, HA-es-ZnPP shows fluorescence quenching, and ^1^O_2_ generation does not occur under light irradiation. However, it is extensively generated when the micelle is disrupted, and ZnPP is released (Figure 4). On the other hand, the micelle formation imposes a prolonged circulation time and tumor-selective accumulation upon HA-es-ZnPP based on the EPR effect (Figure 6). Consequently, a remarkable anticancer PDT effect is achieved both in vitro (Figure 5) and in vivo (Figure 7), suggesting the potential of HA-es-ZnPP as a candidate anticancer nanomedicine for PDT.

## Data Availability

The data presented in this study are available on request from the corresponding author.

## Supplementary Materials

The following are available online at https://www.mdpi.com/2075-4426/11/2/136/s1, Figure S1: Tumor environment-responsive hyaluronan conjugated zinc protoporphyrin for targeted anticancer photodynamic therapy.
