# Supplementary material for: Tumor Environment-Responsive Hyaluronan Conjugated Zinc Protoporphyrin for Targeted Anticancer Photodynamic Therapy
Source: J Pers Med. 2021 Feb 17;11(2):136. doi: 10.3390/jpm11020136 (PMC7922489; doi:10.3390/jpm11020136)
Supplement: Supplementary file 1 [file jpm-11-00136-s001.pdf]

Journal of *Personalized Medicine*

MDPI

Research Article

Tumor Environment-Responsive Hyaluronan Conjugated Zinc Protoporphyrin for Targeted Anticancer Photodynamic Therapy

Supplemental data

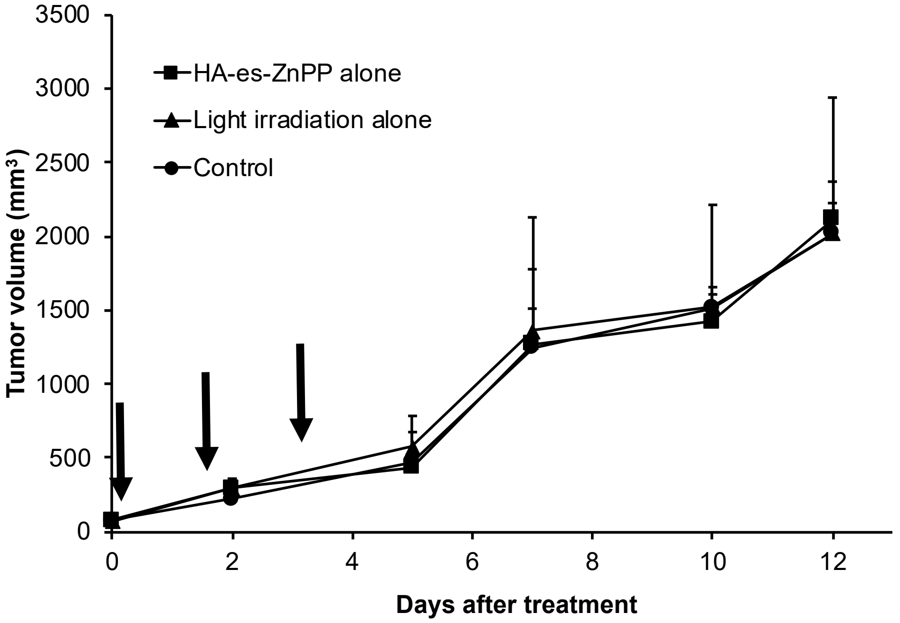

Figure S1. HA-es-ZnPP alone or light irradiation alone did not significant suppression of growth of mouse sarcoma S180 solid tumors. The dosing of HA-es-ZnPP and light irradiation, as well as therapeutic protocol were the same as that shown in Figure 7. Three injections were carried out for HA-es-ZnPP. In light irradiation alone group, physiological saline was injected instead of HA-es-ZnPP. Arrows indicate injection of drugs or light irradiation. Data are mean ± SD.
